# The Application of Artificial Intelligence and Machine Learning in Left Ventricular Assist Device Implantation: A Systematic Review

**DOI:** 10.1111/aor.15025

**Published:** 2025-06-02

**Authors:** Usama Hussain, Wing Kiu Chou, Abhinav Balasubramanian, Jamolbi Rahmatova, Lydia Wilkinson, Arian Arjomandi Rad, Ioannis Dimarakis, Antonios Kourliouros

**Affiliations:** ^1^ Department of Internal Medicine Royal Berkshire Hospital NHS Foundation Trust Reading UK; ^2^ Department of Cardiothoracic Surgery, Oxford Heart Centre, John Radcliffe Hospital Oxford University NHS Foundation Trust Oxford UK; ^3^ Mid and South Essex NHS Foundation Trust Essex UK; ^4^ Department of Internal Medicine United Lincolnshire Hospitals NHS Trust Lincoln UK; ^5^ Department of Cardiothoracic Surgery Maastricht University Medical Center, University of Maastricht Maastricht the Netherlands; ^6^ Division of Cardiothoracic Surgery University of Washington Medical Center Seattle Washington USA

**Keywords:** artificial intelligence, LVAD, machine learning, mechanical circulatory support

## Abstract

**Background:**

This systematic review evaluates the current evidence pertaining to the application of artificial intelligence (AI) and machine learning (ML) in left ventricular assist device (LVAD) implantation. Specifically, the potential of AI/ML in risk stratification, predicting complications, and improving patient outcomes is explored, whereas also identifying key challenges and elucidating avenues of future research.

**Methods:**

A comprehensive search was conducted across EMBASE, MEDLINE, Cochrane, PubMed, and Google Scholar databases to identify studies on AI/ML in LVAD implantation up to March 2024. Articles were selected if they utilized AI/ML techniques in LVAD settings and met predefined criteria. A total of 17 studies were included after a rigorous screening and appraisal process.

**Results:**

The included studies highlighted the use of ML in five main areas: (1) mortality prediction, where ML models demonstrated higher accuracy compared to traditional models; (2) adverse event prediction, including aortic regurgitation and suction events; (3) myocardial recovery, with ML models outperforming traditional stratification methods; (4) deciphering thrombosis risk, with ML identifying key predictors such as younger age and higher BMI; and (5) right ventricular failure prognostication, within which ML models leveraged hemodynamic and imaging data for superior prediction accuracy. Despite such prevalent advances, challenges including data heterogeneity, lack of causality, and limited generalizability persist.

**Conclusion:**

AI and ML possess transformative potential in optimizing LVAD management, offering both advanced prediction of commonly encountered risk occurrence and personalized care respectively. However, identified issues in AI/ML application, including data interpretability, dataset diversity, and integration into clinical workflows, must be addressed in order to enhance their broader adoption and impact.

## Graphical abstract

This systematic review explores the application of artificial intelligence and machine learning in left ventricular assist device implantation, highlighting their superior accuracy in predicting mortality, adverse events, myocardial recovery, thrombosis risk, and right ventricular failure. Despite promising outcomes, challenges like data heterogeneity, interpretability, and clinical integration remain, necessitating further research for broader adoption.

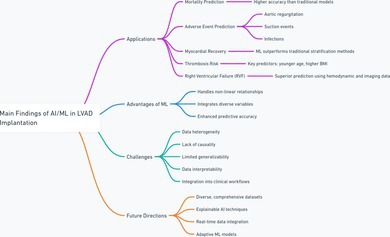

## 1 Introduction

Heart failure remains a global challenge with an ever‐growing prevalence, considerable disease burden, and significant morbidity and mortality [1]. The prognosis for end‐stage disease is poor, and despite advancements in management, patients refractory to guideline‐directed medical therapy for heart failure may require advanced therapies, including left ventricular assist device (LVAD) implantation and heart transplantation [2]. LVADs are valuable to consider when appropriate in the context of advanced heart failure and as a bridge to heart transplantation, particularly given the limited donor pool and the concurrent growing demand [3]. LVAD offers device‐based mechanical circulatory support with validated real‐world improvements in both myocardial recovery and functional capacity, whereas also increasing the 5‐year survival of advanced HF patients [4]. Furthermore, large multicenter trials have suggested that the incidence of event‐free survival and prognosis did not vary significantly between LVAD as a bridge to transplant or as destination therapy alone [5]. Despite the benefits of LVAD, there are well‐recognized risks to patients, significantly postinsertion right ventricular failure (RVF). This remains a direct major cause of mortality, whereas resultant deteriorating renal function and extended length of stay in the ICU both contribute to increased morbidity [6]. The incidence of pump thrombosis (PT) postimplantation remains an important risk, although it has significantly improved in recent years, occurring in 1.4% of patients receiving a HeartMate 3 with a centrifugal pump [7]. Such aforementioned risks highlight the paramount importance of early risk stratification in order to act proactively and mitigate the incidence of detrimental events.

The traditional use of statistical models possesses limitations in analysis due to predominantly utilizing linear comparisons. However, the use of artificial intelligence (AI) and machine learning (ML) enables nonlinear relationships with a more diverse range/type of variables to be identified and enacted upon. This in turn enables the conduction of broader analyses which possess superior accuracy; therefore, aiding in the construction of more comprehensive risk models [8]. The application of such analyses can enable earlier recognition of high‐risk patients and assist decision‐making. In recent years, ML models such as the MARKER‐HF risk score possessed an area under the curve (AUC) of 0.88 and were predictive across the full spectrum of risk in patients presenting with heart failure [9]. ML algorithms built upon the landmark INTERMACS registry have also demonstrated superior discriminative value in their prediction of myocardial recovery following LVAD implantation [10].

Previously published work reviewing the application of ML and LVAD has suggested promising positive predictive value of RVF post‐LVAD insertion [11]. The study, however, omits the evaluation of ML models in predicting other study endpoints after LVAD deployment, such as mortality, myocardial recovery, and other adverse events such as PT. The full utility and application of AI and ML in investigating the impact of LVAD therapy pre, peri‐, and postoperatively has yet to be clarified.

This review aims to systematically evaluate the currently available evidence, investigating the use of AI and ML in the field of LVAD therapy whereas also identifying associated challenges. Based on the challenges recognized, recommendations to LVAD management will be provided alongside a knowledge base for future research in the field of ML and LVAD implantation.

## 2 Methods

### 2.1 Literature Search Strategy

A systematic review was conducted in accordance with the Preferred Reporting Items for Systematic Reviews and Meta‐Analyses (PRISMA) statement and the Cochrane Collaboration published guidelines. EMBASE, MEDLINE, Cochrane, PubMed, and Google Scholar were searched for original articles, which discussed the application of AI or ML in LVAD implantation from inception to March 2024. A priori protocol was devised for the following study, which is available upon request. Applied search terms included “artificial intelligence,” “machine learning,” “neural networks,” “decision trees,” “deep learning,” and “ventricular assist device.” The entire search criteria, which were utilized across all databases, are shown in Appendix 1. Further articles were identified through a manual search of the references lists of articles found through the original search and use of the “related articles” function on MEDLINE. The only limits enforced were the aforementioned time frame and English language.

### 2.2 Study Inclusion and Exclusion Criteria

All original articles were included if they reported the use of AI and ML applications in LVAD. Studies were excluded from the review if: (1) inconsistencies in the data impeded extraction of data, (2) the study was performed in an animal model, (3) there was no mention of LVAD use in participants, (4) there were no AI or ML techniques applied, and (5) less than 10 participants were included in the study. Reviews, editorials, case reports, abstracts from meetings, and preclinical studies were also excluded. By following the aforementioned criteria, two reviewers (W.K.C. and U.H.) independently selected articles for further assessment following title and abstract review. A third independent reviewer (A.B.) resolved any disagreements between the two reviewers. Potentially eligible studies were then retrieved for full‐text assessment. The software used for the described process was Covidence (Melbourne, Australia).

### 2.3 Data Extraction and Critical Appraisal of Evidence

All full texts of retrieved articles were read and reviewed by two authors (U.H and W.K.C.) with a unanimous decision made regarding the inclusion or exclusion of studies. When there was disagreement, the final decision was made by a third reviewer (A.B.). Using a preestablished protocol, the following data were extracted: first author, study design, study country, study population, ML algorithm, and primary study outcomes. A data extraction sheet for this review was developed and pilot‐tested using three randomly selected included studies and subsequently was refined accordingly. Data extraction was performed by two review authors (W.K.C. and U.H.) who carried out the process in duplicate on two separate extraction sheets. Correctness of the tabulated data was validated by a third author (A.B.) who evaluated both extraction sheets and assessed full texts where incongruences existed.

### 2.4 Machine Learning Algorithms and Predictive Analytics

A variety of ML algorithms were utilized across the included studies in this review, each designed to address specific challenges in LVAD management. ML models, including random forests, gradient‐boosted decision trees (e.g., XGBoost), neural networks, Bayesian networks, and support vector machines, were implemented to analyze complex and nonlinear relationships within diverse datasets. Such algorithms offer distinct advantages over traditional statistical models, thereby enabling the inclusion of multiple variables and interactions which may otherwise be overlooked.

Predictive analytics in the studies typically followed a training‐validation approach, wherein datasets were split into subsets for algorithm training and performance evaluation. Common metrics used to assess predictive accuracy included the area under the receiver operating characteristic curve (AUROC), sensitivity, specificity, and net reclassification improvement. The models were tailored to predict outcomes such as mortality, adverse events, myocardial recovery, thrombosis, and RVF. For example, ensemble methods like XGBoost and random forests excelled in stratifying short‐term risk (e.g., 90‐day mortality), whereas neural networks displayed promise in deciphering patterns for complex outcomes such as myocardial recovery.

Explainable AI techniques, such as Shapley additive explanations (SHAP), were occasionally employed to interpret the importance of input variables, enhancing model transparency and clinical utility. By bridging the gap between computational modeling and practical application, these approaches demonstrate the potential for integrating ML into routine LVAD management.

### 2.5 Risk of Bias

**FIGURE 1:**
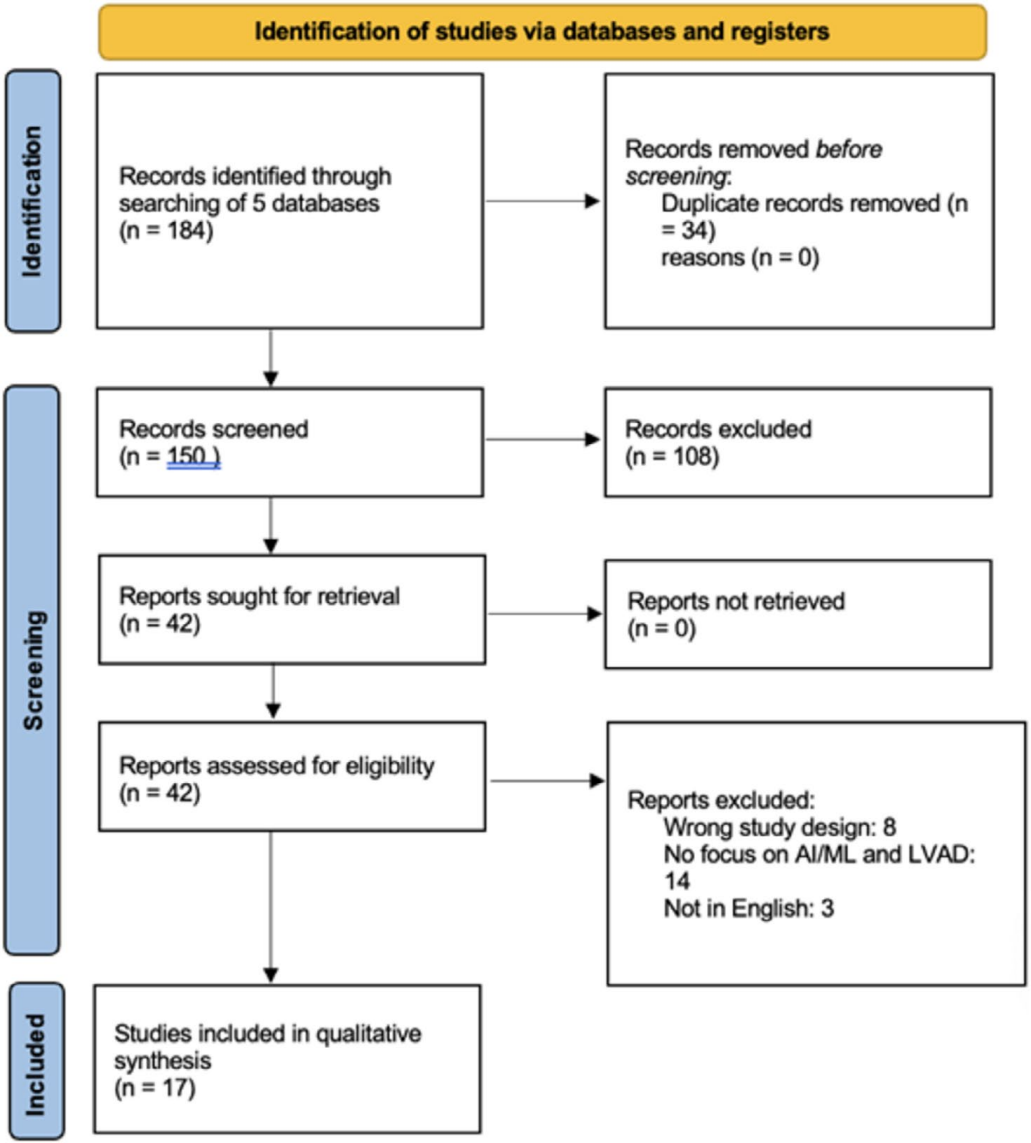
RoB 2 diagram. [Color figure can be viewed at wileyonlinelibrary.com]

The risk of bias in the selected articles was evaluated by two independent reviewers (U.H. and W.K.C.) using an adapted Cochrane Collaboration risk of bias tool (Figure 1). The methodological quality of the studies was assessed based on domains of RoB 2.0: deviation from intended intervention, missing outcome data, measurement of outcome, and selective reporting, classified as low risk of bias, some concerns, or high risk of bias.

## 3 Results

### 3.1 Study Selection

**FIGURE 2:**
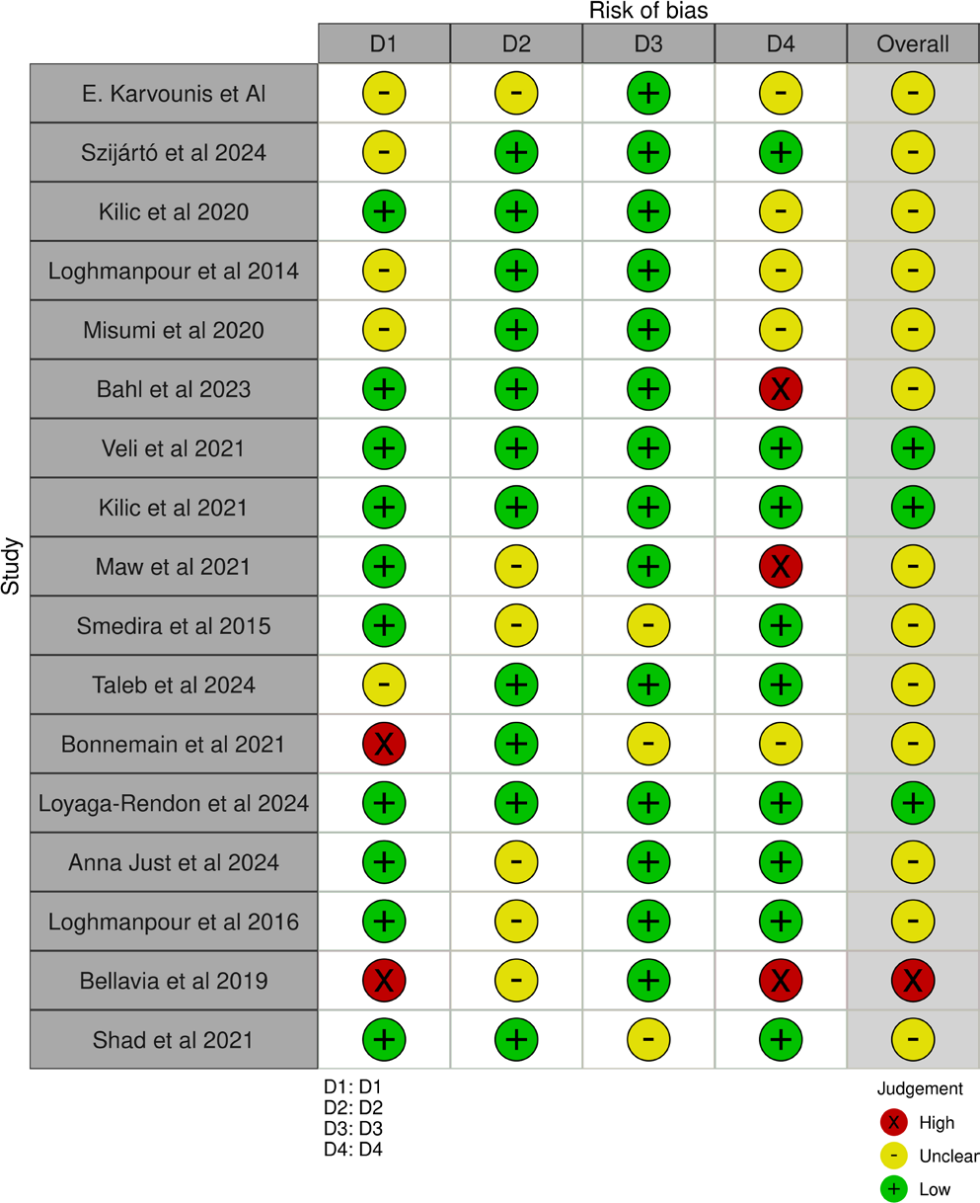
Prisma flowchart. [Color figure can be viewed at wileyonlinelibrary.com]

The literature search identified 184 articles, of which 150 were screened following deduplication and 42 were full‐text reviewed and assessed in accordance with inclusion and exclusion criteria. Following critical appraisal, a total of 17 studies were included in this review. Figure 2 illustrates the entire study selection process. A summary of the studies collected and their respective designs, patient population, country, study aims, and the main reported outcomes are presented in Table 1 [12, 13, 14, 15, 16, 17, 18, 19, 20, 21, 22, 23, 24, 25, 26, 27, 28].

**TABLE 1:** Summary of included studies and their main characteristics.

Study	Population number	Country	Algorithm/model/method of implementation	Study objective	Main reported outcomes
**AI prediction of myocardial recovery**					
Evaggelos Karvounis et al. [12]	49 (TS), 6 (MO), 11 (WE)	Greece, Italy, Belgium	Specialist Decision Support System (SDSS)—treatment support (TS), monitoring (MO), and weaning (WE)	Analysis of SDSS, a web‐based AI tool which assists specialists to design therapy plan for their patients before and after LVAD implantation	Of the 49 in the TS group; 35 had no relevant adverse events; 3 had bleeding episodes and 11 died. From 6 (MO), 1026 patterns have been created, including patterns covering three classes, being normal (1022), ventricular tachycardia (3), and heart failure (1). From the 11 (WE), patterns were derived for recognizing when to wean and not wean, 4 of the 11 were weaned off LVAD
Veli et al. [18]	20 270	USA	Least absolute shrinkage and selection operator (LASSO), Bayesian logistic regression (B‐LR), linear support vector machine, gradient‐boosted decision tree, neural network, and random forest with 10‐fold cross‐validation	To determine whether the use of ML‐based models predict LVAD patients with myocardial recovery resulting in pump explant	ML models achieved an AUC of 0.813–0.824 in the validation dataset, outperforming logistic regression‐based risk scores
**AI‐based risk stratification**					
*Mortality*					
Kilic et al. [14]	16 120	USA, Canada	Extreme gradient boosting or XGBoost	Compare ML and linear regression‐based post‐LVAD risk prediction models for 90‐day and 1‐year overall mortality	C‐index was improved with the ML model (90‐day: 0.707 vs. 0.740 and 1‐year: 0.691 vs. 0.714; each *p* < 0.001). Net reclassification index analysis demonstrated improvement of 48.8% and 36.9% for 90‐day and 1‐year mortality, respectively, with ML (each *p* < 0.001). ML can improve risk model performance as compared to linear regression models
Loghmanpour et al. [15]	8050	USA	Bayesian networks—supervised machine learning	To develop and validate a predictive model for 90‐day survival of patients with continuous‐flow LVAD using Bayesian networks, and compare against HeartMate 2 Risk Score (HMRS)	Postimplant mortality at 90‐day and 1 year, achieving accuracies of 90 and 83, Kappa values of 0.37 and 0.45, and AUC of 82 and 80 respectively. This was in comparison to the HMRS with an AUC of 57% and 60% at 90‐days and 1‐year, respectively
Loyaga‐Rendon et al. [24]	3853	USA	Elastic net and neural network (NN) models	The study aims to develop a predictive model for 1‐year mortality in patients with end‐stage heart failure who received a HeartMate 3 (HM3) left ventricular assist device (LVAD) using machine learning (ML) algorithms	The elastic‐net algorithm with all variables had an AUC of 0.70, similar to the logistic regression model (AUC: 0.72). With selected variables, the AUC improved to 0.72. The neural network model, when supplied with all variables, had an AUC of 0.69. With selected variables, the AUC decreased to 0.65
*Right ventricular failure*					
Szijártó et al. [13]	29	Hungary, USA	Multiple: multilayer perceptron (MLP), linear regression, K‐nearest neighbors, support vector machines, random forest	To develop an ML‐based method that can reconstruct the RV pressure curve based on the peak RV pressure	MLP achieved the best performance among the algorithms. The MLP and the RV reference curve‐based estimation showed good agreement with the invasive RV pressure curves (mean bias: −0.38 mmHg and −0.73 mmHg, respectively), whereas the LV reference curve‐based estimation exhibited a high mean bias (+3.93 mmHg)
Loghmanpour et al. [26]	10 909	USA	Tree‐augmented naïve Bayesian architecture	To develop a Bayesian‐based prognostic model of RVF following implantation of a continuous‐flow LVAD, and other previously published risk scores	Three separate Bayesian models for acute, early, and late RVF substantially outperformed the existing risk scores in their ability to predict the risk of RV failure. The accuracy of all Bayesian models was between 91% and 97%, with an AUC between 0.83 and 0.90, sensitivity of 90%, and specificity between 98% and 99%, significantly outperforming previously published risk scores
Bahl et al. [17]	19 595	USA	Boosted decision tree machine learning algorithm (LightGBM) with Shapley additive explanations (SHAP)	To derive novel insights into preimplant patient factors associated with right heart failure (RHF) after LVAD implantation using explainable machine learning methods	Out of 19 595 patients, 19.1% developed severe RHF at 30 days. Thirty top predictors of RHF were identified
Taleb et al. [22]	1125	USA	Bootstrap imputation and adaptive least absolute shrinkage and selection operator variable selection techniques	To derive and validate a risk model to predict RVF after LVAD implantation	The calculator achieved a *C* statistic of 0.75 in the derivation cohort and 0.73 in the validation cohort. Cumulative survival was higher in patients composing the low‐risk group (estimated < 20% RVF risk) compared with those in the higher risk groups. The STOP‐RVF risk calculator exhibited a significantly better performance than commonly used risk scores
Bellavia et al. [27]	74	Italy, USA	Linear support vector machines and Naïve Bayes algorithms	To identify most accurate predictors of RVF among clinical, biological, and imaging markers, assessed by agreement of different supervised machine learning algorithms	Michigan risk score combined with central venous pression assessed invasively and apical longitudinal systolic strain (sS) of the RV free wall were the most significant predictors of acute RVF (AUC = 0.95, by the Naïve Bayes), whereas the RV free wall sS of the middle segment right atrial strain (QRS‐synced) and TAPSE were the most significant predictors of chronic RVF (AUC = 0.97, according to Naïve Bayes)
Shad et al. [28]	721	USA	Video‐based deep learning	The primary aim is to develop and validate a deep learning model to predict postoperative right ventricular (RV) failure using video‐based echocardiography data	The study demonstrated the potential of video‐based deep learning models in predicting postoperative RV failure, showing that the AI system performed comparably or better than clinical risk scores and human experts
*Others: infection, bleeding, thrombosis, suction, aortic regurgitation*					
Kilic et al. [14]	568	USA	Various machine learning algorithms including hierarchical clustering based on the longest common subsequence	To employ machine learning algorithms to visualize and assess sequences of adverse events (AEs) after LVAD implantation	Identified distinct patterns and relationships among AEs. Generated five distinct clusters of patients with different AE profiles. Bleeding and infection were the most common AEs, constituting approximately two‐thirds of all AEs. High transition probabilities were observed from bleeding to subsequent bleeding (31%) and from infection to subsequent infection (34%)
Smedira et al. [21]	10 208	USA	Machine learning, nonparametric random forests for survival	To verify whether the increase in pump thrombosis risk occurred nationwide, determine if risk continued to rise, leveled at a new rate, or decreased. To identify patient and implant procedure predictors of pump thrombosis, to explore variability in risk of pump thrombosis among implanting centers, and to assess the effect of pump thrombosis on subsequent mortality	A total of 995 pumps thrombosed, with risk peaking within weeks of implant. The risk‐adjusted increase in pump thrombosis began in 2010, reached a maximum in 2012, and then plateaued at a level that was 3.3‐times higher than pre‐2010. pump exchange, younger age, and larger body mass index were important predictors, and institutional variability was largely explained by implant date, patient profile, and duration of support. The probability of death within 3 months after pump thrombosis was 24%
Maw et al. [20]	38	Austria	A single feature algorithm (SFA), a CART decision tree (CDT), and an adaptively boosted ensemble of decision trees (ABE)	To use ML to automatically detect suction events without additional sensors using a database of patient data from those implanted with the Medtronic HVAD device	A single feature classifier could perform on a similar level to more complex algorithms on a per‐snapshot basis (test sensitivity: 100% specificity: 95.5%). An adaptively boosted tree ensemble classifier managed to achieve higher accuracy on a per‐beat basis, but showed signs of overfitting with a reduction in performance
Misumi et al. [16]	13	Japan	Supervised machine learning involving an ensemble classifier and tenfold stratified cross‐validation	To determine whether acoustic signal analyses and machine‐learning modeling could be used to detect serious aortic regurgitation (AR) occurring after LVAD implantation	The most useful variables for predicting concomitant AR were the amplitude of the first harmonic, LVAD rotational speed during intermittent low speed (ILS), and the variation in the amplitude during normal rotation and ILS. The predictive accuracy and area under the curve (AUC) were 91% and 0.73, respectively
Bonnemain et al. [23]	0 patients, simulated data	Switzerland	Deep neural network (DNN)	To evaluate whether a DNN approach could be used to predict parameters of LV systolic function under LVAD support. The specific objective is to assess LV hemodynamics under device assistance, which could be helpful for a better understanding of LV‐LVAD interactions, and for therapeutic optimization	The DNN‐predicted Emax, lv with a mean relative error of 10.1%, and all other parameters of LV function with a mean relative error of < 13%. The framework was then able to retrieve a number of LV physiological variables (i.e., pressures, volumes, and ejection fraction) with a mean relative error of < 5%
Anna Just et al. [25]	137	Germany	AI‐based automated software tool (U‐net) for image segmentation	To investigate the predictive value of body composition parameters assessed by artificial intelligence‐based analysis in patients receiving long‐term mechanical circulatory support	The study found that body composition parameters, such as visceral adipose tissue (VAT) and sarcopenia, have predictive value for patient outcomes post mechanical circulatory support. Specifically, nonobese and nonsarcopene individuals showed significant improvements in 6‐min walk distance and quality of life after 6 months

### 3.2 The Role of AI in Predicting Myocardial Recovery From LVAD

Two studies explored the ability of AI to predict myocardial recovery after LVAD implantation as a primary outcome, whereas a third study analyzed this as a secondary outcome [12, 18, 23]. One study involving 20 270 patients used 98 variables to train ML models and outperformed conventional stratification scores in identifying patients likely to experience myocardial recovery [18]. Another study focused on 11 patients and used ML models trained on 70 variables to identify 32 patterns associated with potential myocardial recovery, enabling four patients to be weaned off LVADs [12].

### 3.3 The Role of AI in the Complications of LVAD Implantation

#### 3.3.1 Risk Stratification

Nine studies investigated the use of AI/ML‐based models for risk stratification in LVAD patients, focusing on mortality and adverse events. Five studies [12, 14, 15, 22, 24] examined mortality outcomes, with a total of 28 870 participants. Commonly measured outcomes included all‐cause mortality, 90‐day mortality, and 1‐year mortality. ML models consistently demonstrated superior accuracy compared to conventional risk models based on linear regression. For instance, Kilic et al. [14] reported significant improvements in AUROC for ML models, particularly for predicting 90‐day and 1‐year mortality.

Four studies [16, 19, 20, 25] assessed adverse events, employing diverse methodologies and outcome measures. One study predicted aortic regurgitation using AI models based on LVAD sound signals [16]. Another study addressed suction events, a common LVAD complication linked to arrhythmias and poorer outcomes, using an AI model trained on expert annotations of cardiac cycles [20]. Other studies used AI to predict adverse events such as infections or analyzed body composition via preoperative CT scans, linking higher adipose tissue levels with increased infection risk and in‐hospital mortality [19, 25].

#### 3.3.2 Thrombosis

Thrombosis‐related outcomes were less frequently studied. In a study by Smedira et al. [21], an analysis of 11 123 HeartMate II devices over 6 years identified 995 cases of PT. ML models assessed 87 preimplant and implant variables and identified that the incidence of pump exchange, younger age, and increased BMI were significant predictors of thrombosis, with an associated 3‐month mortality rate of 24% post‐thrombotic event. More recent technological advancements to LVAD technology have been made since the study by Smedira et al.; however, AI‐based studies on predicting pump thromboses in the newer third‐generation LVADs have not been identified in our search [7]. Some studies linked suction as a potential risk factor for thrombosis, correlating it with arrhythmias and poorer 90‐day outcomes [20].

#### 3.3.3 Mortality

The ability of ML models to outperform traditional models in predicting mortality outcomes was mixed. Loyaga‐Rendon et al. [24] evaluated two algorithms and found that complex ML models did not consistently outperform standard techniques. Conversely, Kilic et al. [14] demonstrated significant improvements in discriminatory performance with ML models for both 90‐day and 1‐year mortality. Loghmanpour et al. [15] showed that Bayesian network risk scores were reproducible and clinically relevant at multiple time points, although their predictive power diminished over time. Interestingly, the AUROC was higher for predicting 90‐day mortality compared to 1‐year mortality for both logistic regression (LR) and ML models, suggesting that preimplant risk factors may be less predictive of long‐term survival post‐LVAD surgery.

#### 3.3.4 Right Ventricular Failure

Four studies [17, 22, 27, 28] investigated RVF outcomes. A supervised ML‐based STOP‐RVF score was effective in identifying patients at risk of RVF following LVAD implantation, although adding intraoperative characteristics did not enhance predictive accuracy [22]. The use of preoperative data was particularly effective for stratifying RVF risk, influencing both patient selection and treatment decisions. Three studies [22, 27, 28] utilized video‐based deep learning approaches, with Bellavia et al. [27] finding that regional right ventricular longitudinal strain and central venous pressure were significant predictors of RVF.

## 4 Discussion

**FIGURE 3:**
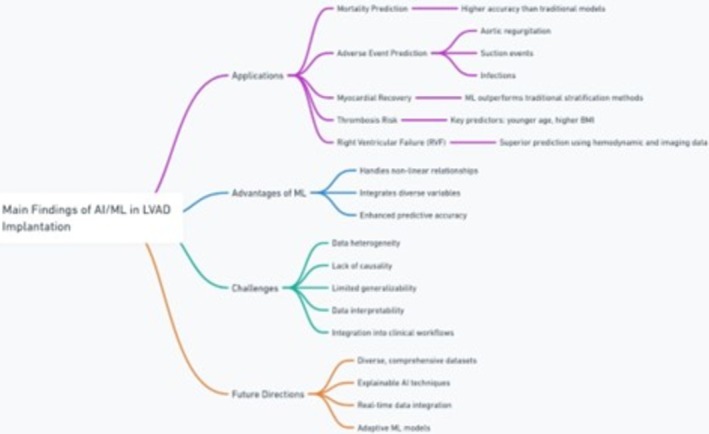
Main findings of AI/ML in LVAD implantation. [Color figure can be viewed at wileyonlinelibrary.com]

The use of ML models for risk stratification in LVAD patients shows considerable promise, consistently outperforming traditional approaches across various outcomes, including mortality, adverse events such as PT and RVF, and therapeutic outcomes such as myocardial recovery (Figure 3).

### 4.1 The Role of AI in Predicting Myocardial Recovery From LVAD

Myocardial recovery prediction remains a challenging area, yet ML models have demonstrated early successes in otherwise unassessed areas. A study involving 20 270 patients illustrated that ML could outperform traditional stratification scores in identifying patients with the potential for myocardial recovery, leveraging a large set of 98 variables [18]. A further study highlighted the capability of ML to identify patterns within cohorts of a reduced sample size that subsequently determined eligibility for LVAD weaning [12]. These results illustrate ML's potential in recognizing nuanced patterns in complex datasets and suggest that integrating AI into clinical decision‐making could enhance personalized patient care with resulting positive outcomes. However, the small sample size and lack of specific myocardial recovery data limit the conclusions that can be drawn. Larger prospective trials with a more heterogeneous sample are necessary to validate these findings and ensure their applicability in real‐time decision‐making, particularly in the context of predicting long‐term recovery.

### 4.2 The Role of AI in the Complications of LVAD Implantation

ML‐based models demonstrated high sensitivity and accuracy, particularly in predicting short‐term outcomes such as 90‐day and 1‐year mortality. Kilic et al. [14] also demonstrated that ML significantly improved discriminatory performance for 90‐day mortality, corroborating the suggestion that preoperative factors play a critical role in early postimplant outcomes. Therefore, indicating that ML models could be especially useful for early postimplant risk assessment and patient management.

In terms of predicting adverse events, ML models displayed distinct advantages in analyzing complex, nonlinear relationships which traditional methods struggle to interpret. Techniques such as AI‐based sound analysis for predicting aortic regurgitation and classifiers trained on cardiac cycle annotations for predicting suction complications underscore the ability of ML to integrate diverse physiological data [16]. Such aforementioned findings therefore underscore the diverse applicability of ML models in predicting adverse events beyond mortality, and present a novel noninvasive technique such as hemodynamic signals to inform a more comprehensive risk assessment. Our review also identified that the use of AI in predicting aortic regurgitation was only seen in one study [16], and no study looked at AI‐based prediction of driveline infections. There is scope for future studies to explore AI applications in predicting these concerning complications of LVAD insertions.

For thrombosis‐related outcomes, ML models provided insights into risk factors which were not easily detectable through analysis via conventional methods. Smedira et al. [21] presented that predictors such as younger age, higher BMI, and pump exchange were significantly linked to thrombosis risk in a directly proportional manner, thus highlighting the importance of early detection and optimizing LVAD settings. The link identified between suction and thrombosis also suggests that ML models could be instrumental in real‐time LVAD adjustments to reduce the resulting thrombotic risk; an important association when considering the effect of pump head thrombosis is reduced LVAD function and potential vessel occlusion if dislodged. Furthermore, our review did not identify any AI‐based assessment on outflow graft position/anatomy and associated outcomes. Further studies focusing on this could better inform risk prediction, for example, thrombogenesis owing to the variable hemodynamic characteristics in different positions.

Regarding mortality prediction, ML models provided a more nuanced understanding of patient outcomes compared to traditional methods. Variables like the MELD score and right atrial pressure emerged as critical predictors that were often overlooked by conventional models, highlighting the strength of ML in capturing complex health dynamics. Kilic et al. [14] and Loghmanpour et al. [15] showed that although ML models perform well in short‐term mortality prediction, their predictive power for long‐term outcomes diminishes, likely due to evolving patient conditions that are not dynamically accounted for in real time. This points to the need for adaptive ML models that can update predictions based on real‐time data, thereby enhancing their utility throughout the patient care continuum.

In RVF prediction, ML showed significant potential by offering dynamic and detailed assessments that traditional models lack. Video‐based deep learning approaches, such as those used by Bellavia et al. [27], enhanced RVF prediction accuracy through the analysis of regional strain and hemodynamic parameters. This method's ability to capture the dynamic nature of right ventricular function provides a more individualized assessment, which can improve patient selection and postimplant care strategies. However, limitations such as operator bias and inconsistent definitions of RVF across studies must be addressed through standardized diagnostic criteria and multi‐institutional data collection to improve model robustness. Despite the central role of anticoagulation and antiplatelet therapy in LVAD patient management, our review did not identify any studies applying AI or ML techniques to this domain. This may reflect challenges in data standardization, therapeutic variability, or the nascent stage of applying predictive models in pharmacological monitoring. Nevertheless, given the known risks of thromboembolism and bleeding, this represents an important unmet need in the literature and a promising direction for future AI‐driven research.

The integration of ML models into clinical practice for LVAD patient management holds transformative potential. By accurately predicting risk, guiding personalized interventions, and providing real‐time insights into patient conditions, ML models can significantly improve outcomes for patients with advanced heart failure. To develop this potential, future research must focus on building diverse and comprehensive datasets, incorporating both real‐time clinical data, and developing explainable AI techniques. Additionally, partnerships with multi‐institutional consortia could enhance dataset diversity, whereas clinical trials exploring real‐time ML applications could offer critical data on their utility in routine practice.

Ultimately, the incorporation of ML into LVAD management is a promising pathway for furthering personalized patient care by developing adaptive and effective learning mechanisms. Addressing the key limitations of data quality, explainability, and diversity will be crucial in furthering ML models as reliable and equitable tools for clinicians, ensuring that these advanced technologies contribute meaningfully to improved patient outcomes.

### 4.3 Limitations

Despite the promising performance of ML models in LVAD management, several challenges must be addressed for successful clinical integration. The variability in study aims, quality of data, and outcome measures limits the ability to generalize the findings across heterogeneous patient populations. Many studies relied on small, homogeneous cohorts, which raise significant concerns about the model's applicability to non‐White populations. Aside from the study by Taleb et al. [22] the lack of diversity in the combined participant population introduces biases that could perpetuate healthcare disparities, particularly when ML models are applied in real‐world clinical settings.

Of the 17 studies included, four demonstrated bias in one or more domains, whereas most were assessed as having unclear risk, and only three were rated as overall low risk of bias. This heterogeneity in methodological quality suggests that, although some findings are supported by robust evidence, the overall conclusions of this review should be interpreted with caution, particularly in areas where data are limited or the risk of bias is uncertain.

Another critical barrier to adoption is the complexity of many ML models, particularly deep learning algorithms, which often act as “black boxes.” Clinicians require interpretable and actionable insights to integrate these models into their decision‐making processes confidently. The absence of explainable AI techniques and user‐friendly interfaces prevents healthcare professionals from understanding and trusting the model outputs. Future development of hybrid models which blend ML and traditional scoring systems could help bridge this gap by combining the interpretability of conventional methods with the predictive power of ML.

Clinically relevant post‐LVAD outcomes such as ischemic and hemorrhagic stroke, gastrointestinal bleeding, device‐related infections, psychosocial effects, long‐term outcomes in destination therapy, and bridging success to transplantation were largely underrepresented in the wider literature. The role of ML algorithms in deciding outflow graft positioning and prediction of outcomes has yet to be clarified. The absence of such salient outcomes reflects both the evolving nature of this field and the scope of our search.

Furthermore, the literature search concluded in March 2024. Although this timeframe captured the rapid evolution of AI in LVAD management up to that point, it is likely that additional studies—particularly those addressing third‐generation devices and real‐time predictive models—have since emerged. This underlines the need for future reviews to incorporate more recent data and reassess the breadth of AI applications in this space.

## 5 Conclusion

This systematic review highlights the potential of ML and AI to enhance the prediction of clinical outcomes, mortality, and adverse events in patients receiving LVADs. Although traditional risk models have offered useful insights to date, ML‐based models consistently outperform them by capturing nonlinear relationships and incorporating a broader array of variables. ML models have also demonstrated promise in predicting the occurrence of RVF, PT, myocardial recovery, and overall mortality, thereby providing clinicians with ever‐evolving tools to execute early risk stratification and subsequently guide decision‐making.

## Author Contributions

Usama Hussain, Wing Kiu Chou, Abhinav Balasubramanian, Jamolbi Rahmatova, Lydia Wilkinson, Arian Arjomandi Rad, Ioannis Dimarakis, and Antonios Kourliouros contributed to the conception and design of the article, data collection, original writing, visualisation, and final approval. Usama Hussain, Wing Kiu Chou, and Abhinav Balasubramanian were responsible for data analysis and visualisation. Arian Arjomandi Rad, Ioannis Dimarakis and Antonios Kourliouros supervised the project. All authors contributed to the writing, critical revision of the article, and final approval of the manuscript.

## Conflicts of Interest

The authors declare no conflicts of interest.

## Data Availability

Data collection form and search results are available on enquiry to the corresponding author.

## Appendix 1

**Table:**

1	artificial intelligence.ti,ab.	11043
2	machine learning.ti,ab.	15512
3	neural networks.ti,ab.	6220
4	decision trees.ti,ab.	640
5	deep learning.ti,ab.	7544
6	1 or 2 or 3 or 4 or 5	36212
7	heart failure.ti,ab.	97644
8	assist device.ti,ab.	7840
9	LVAD.ti,ab.	3636
10	left‐ventricular assist device.ti,ab.	4685
11	ventricular assist device.ti,ab.	6000
12	7 or 8 or 9 or 10 or 11	104146
13	6 and 12	184

# Artificial Intelligence and Related Methods

("Artificial Intelligence"[MeSH Terms] OR "Artificial Intelligence"[tiab] OR

"Machine Learning"[MeSH Terms] OR "Machine Learning"[tiab] OR

"Neural Networks, Computer"[MeSH Terms] OR "neural networks"[tiab] OR

"Decision Trees"[tiab] OR

"Deep Learning"[tiab])

AND

# Heart Failure and Ventricular Assist Devices

("Heart Failure"[MeSH Terms] OR "heart failure"[tiab] OR

"Ventricular Assist Devices"[MeSH Terms] OR "assist device"[tiab] OR

"LVAD"[tiab] OR

"left‐ventricular assist device"[tiab] OR

"ventricular assist device"[tiab])
